# Mitochondrial DNA disorders: from pathogenic variants to preventing transmission

**DOI:** 10.1093/hmg/ddab156

**Published:** 2021-06-24

**Authors:** Tiago M Bernardino Gomes, Yi Shiau Ng, Sarah J Pickett, Doug M Turnbull, Amy E Vincent

**Affiliations:** 1 Wellcome Centre for Mitochondrial Research, Translational and Clinical Research Institute, Newcastle University, Newcastle upon Tyne NE2 4HH, UK; 2 NHS Highly Specialised Service for Rare Mitochondrial Disorders, Newcastle upon Tyne Hospitals NHS Foundation Trust, Newcastle upon Tyne NE2 4HH, UK

## Abstract

Mitochondrial DNA (mtDNA) disorders are recognized as one of the most common causes of inherited metabolic disorders. The mitochondrial genome occurs in multiple copies resulting in both homoplasmic and heteroplasmic pathogenic mtDNA variants. A biochemical defect arises when the pathogenic variant level reaches a threshold, which differs between variants. Moreover, variants can segregate, clonally expand, or be lost from cellular populations resulting in a dynamic and tissue-specific mosaic pattern of oxidative deficiency. MtDNA is maternally inherited but transmission patterns of heteroplasmic pathogenic variants are complex. During oogenesis, a mitochondrial bottleneck results in offspring with widely differing variant levels to their mother, whilst highly deleterious variants, such as deletions, are not transmitted. Complemented by a complex interplay between mitochondrial and nuclear genomes, these peculiar genetics produce marked phenotypic variation, posing challenges to the diagnosis and clinical management of patients. Novel therapeutic compounds and several genetic therapies are currently under investigation, but proven disease-modifying therapies remain elusive. Women who carry pathogenic mtDNA variants require bespoke genetic counselling to determine their reproductive options. Recent advances in *in vitro* fertilization techniques, have greatly improved reproductive choices, but are not without their challenges. Since the first pathogenic mtDNA variants were identified over 30 years ago, there has been remarkable progress in our understanding of these diseases. However, many questions remain unanswered and future studies are required to investigate the mechanisms of disease progression and to identify new disease-specific therapeutic targets.

## Introduction

It is 40 years since sequencing of the human mitochondrial genome was first reported, showing that mitochondrial DNA (mtDNA) is a small (16.6 kb) circular genome (1,2) containing only 37 genes—13 protein-encoding (all oxidative phosphorylation (OXPHOS) subunits), 22 tRNA genes and 2 rRNA genes. All other mitochondrial proteins (~1100) are nuclear-encoded, translated in the cytoplasm and transported into the mitochondria (3). In 1988, the first mtDNA pathogenic variants, causing human disease, were described—single, large-scale mtDNA deletions (4) and a single-nucleotide variant causing Leber’s Hereditary Optic Neuropathy (LHON) (5).

This review concentrates on the advances in our understanding of clinical mtDNA genetics over the last 30 years. Mitochondrial genetics is complex, due to the interplay between mitochondrial and nuclear genomes and the peculiar genetics of mtDNA. In the absence of animal models, until recently, that reflect the biology and phenotype of mtDNA disease, much of our understanding comes from the study of patients. These have shown how mtDNA diseases are relatively common and highlighted the difficulties in the diagnosis and management of patients (6). Whilst there have been considerable advances there are still many challenges.

### Pathogenic mtDNA variants are an important cause of disease

Since their first description in 1988, there has been increasing recognition of the importance of mtDNA variants in human disease. Whilst several studies suggest that mtDNA variants are risk factors for a growing number of common diseases (7,8), this review will focus on those primarily caused by pathogenic mtDNA variants.

One of the great challenges of mtDNA disease is the varied clinical presentation in terms of age of onset, from the neonatal period to late adult life, and combination of clinical features (9). Tissues and organs heavily dependent upon OXPHOS (e.g. skeletal and cardiac muscles and brain) are predominantly affected, but any tissue can be involved. Historically, patients were categorized into specific syndromes such as Kearns-Sayre syndrome (10) or mitochondrial encephalopathy, lactic acidosis, and stroke like episodes (MELAS) (11). However, most patients present with a spectrum of nonspecific features (e.g. fatigue, diabetes or deafness) and mtDNA disease is suspected through clinical pattern recognition and family history of mtDNA maternal inheritance (9). Most patients have involvement of multiple organs and thus diagnosis and care by a multidisciplinary team is required.

This phenotypical variability has made prevalence very difficult to establish. Population studies looking at mtDNA pathogenic variants suggest a surprisingly high frequency (1 in 250 people in some studies) and that most carriers are asymptomatic (12). Studies on the prevalence of mtDNA diseases have shown that they affect about 10 adults per 100 000 of the UK population, with another 10 per 100 000 family members at risk of developing disease (6). Additionally, mitochondrial disease in children is mostly caused by nuclear pathogenic variants (~75%) (13); whereas ~2/3 of affected adults carry a mtDNA variant (6).

### Mitochondrial DNA variants may be homoplasmic or heteroplasmic

The mitochondrial genome exists in multiple copies in most cells—varying from hundreds in some cells to several hundred thousand in human oocytes (9). When all copies are identical, this is called homoplasmy; a mixture of genomes is called heteroplasmy. Some mtDNA diseases are caused by homoplasmic variants—such as LHON, which is caused by one of three common variants affecting complex I subunits (5). However, most pathogenic mtDNA variants are heteroplasmic, and exist in a mixture with wild-type mtDNA—such as the common m.3243A>G variant (14) and single, large-scale mtDNA deletions (15).

### Mitochondrial DNA variants may be inherited or arise *de novo*

Whilst many patients have a family history of mitochondrial disease, a significant number of patients have *de novo* mutations (16). The best example are single, large-scale mtDNA deletions, which are mostly sporadic and represent ~10% of adults with mitochondrial disease (6). Sporadic single-nucleotide variants are also a significant cause of mtDNA disease (16–18) and demonstrate the importance of mtDNA sequencing when mitochondrial disease is suspected.

### MtDNA is maternally inherited

In 1980, mtDNA was found to be maternally inherited by studying familial polymorphic variants (19). Paternal transmission has been suggested, but the presence of large portions of mtDNA transposed into nuclear DNA, so-called nuclear mitochondrial DNA, has raised doubts about these observations (20–22). Certainly, for mtDNA disease, there are no reports of paternal transmission. Therefore, maternal transmission can be one of the first clues for diagnosis. For heteroplasmic mtDNA variants, transmission is complicated by the presence of a genetic bottleneck in early development due to a restriction in the number of mitochondrial genomes repopulating the female germ line (23–26). This means that the level of variant can vary considerably in siblings.

### Threshold and clonal expansion of mtDNA variants

**Figure 1:**
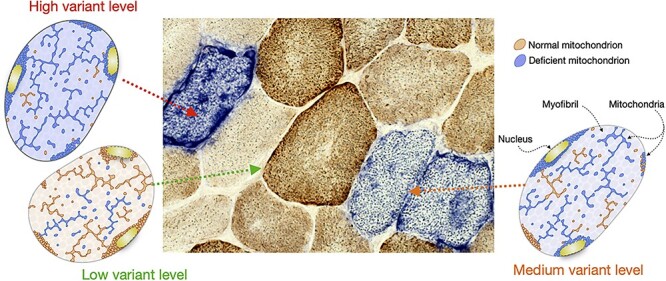
Oxidative phosphorylation deficiency correlates with variant levels of mtDNA pathogenic variants in a tissue mosaic pattern. Tissues from patients with mitochondrial disease present with a mosaic pattern of mitochondrial dysfunction when reacted for cytochrome c oxidase/succinate dehydrogenase (COX/SDH) histochemstry. Mitochondrial DNA molecules are present in hundreds of copies per cell and a variant can be present in any proportion of these; this is termed variant level. When all mtDNA molecules are wild-type or when the mtDNA variant exists at a low level, the cells become brown on COX/SDH treated tissue sections indicating preserved COX activity as a surrogate of normal mitochondrial function. When present at levels exceeding a threshold of biochemical deficiency the mitochondria appear dysfunctional and the cells are blue due to the lack of COX activity. The figure shows an example of a COX/SDH reacted cross-section of skeletal muscle from a patient with mitochondrial disease and the respective schematics for myofibres with low, medium and high levels of variant. Each schematic represents a myofibre in cross section showing the contractile myofibrils (grey circles) in the sarcoplasm and the multiple nuclei (yellow). Mitochondria are distributed between the myofibrils, under the myofibre membrane and around the nuclei, and vary in shape from round to elongated and branched, partially depending on their location. As in the microscopy image, brown indicates normal mitochondria and blue represents those exhibiting respiratory chain deficiency.

One of the earliest observations in patients with mtDNA disease was the presence in tissues (particularly skeletal muscle) of a mosaic pattern of cells with either normal OXPHOS activity or deficiency (Fig. 1) (27). This characteristic finding is still important in the diagnosis of mitochondrial disease but was crucial for our understanding of disease threshold for a particular mtDNA variant. Isolating individual cells and determining the relative level of variant in each cell shows that mtDNA variants are functionally recessive with threshold of mutated to wild-type mtDNA of about 60% for single, large-scale mtDNA deletions up to over 90% for some single-nucleotide variants (28–32). In terms of OXPHOS deficiency, the crucial component is the amount of wild-type mtDNA to support respiration (33).

In heteroplasmic conditions, mtDNA variants clonally expand within cells due to relaxed replication of the mitochondrial genome independent of nuclear DNA replication (34). This means that adjacent cells can have very different loads of mutated mtDNA and therefore a very different biochemical phenotype, leading to a mosaic pattern of deficiency. The exact mechanism of clonal expansion is unknown although there are several hypotheses (35).

### Genetic behaviour of specific types of pathogenic mtDNA variants

#### Homoplasmic mtDNA pathogenic variants

The best-known homoplasmic mtDNA variants are those that cause LHON (36). The variants are present in all tissues but despite this, most patients who develop symptoms have subacute loss of vision due to optic nerve atrophy, highlighting that the phenotype associated with an mtDNA variant can be tissue specific. There is incomplete penetrance and unidentified nuclear genetic and/or environmental factors such as hormonal differences, smoking or alcohol are likely to contribute the disease phenotype (37–40)—whilst ~50% of male carriers of LHON variants develop subacute visual loss, far fewer females develop symptoms (36). Traditionally, this ratio was thought to be close to 5:1, but recently a 3:1 male-to-female ratio was found in a large international LHON population (36,41). This study raises concerns about ascertainment bias towards young men in clinical practice and highlights the need to consider LHON as a differential in older patients and women with acute or subacute visual loss.

#### Heteroplasmic mtDNA pathogenic variants

Whilst all heteroplasmic variants exist in a mixture with wild-type mtDNA, not all behave the same *in vivo.* The level of some variants is stable over time in all tissues, while others are selected against in replicating cells (42,43). Understanding these different behaviours is important for prognostics and reproductive advice. This categorization of heteroplasmic mtDNA variants does not influence disease severity and any variant can cause very severe disease.

#### Heteroplasmic mtDNA pathogenic variants with strong selection in replicating tissues

A good example of this type of mutation is a single, large-scale mtDNA deletion which removes several kb of the mitochondrial genome. Patients may present with a continuum of syndromes including Pearson’s syndrome, a very rare and severe illness in infancy (44); Kearns-Sayre syndrome, a childhood onset disease with prominent neurological involvement; and chronic progressive external ophthalmoplegia developing in late adult life (15). Infants with Pearson’s syndrome have sideroblastic anaemia, pancreatic insufficiency, and failure to thrive but if they survive the initial illness, they then progress to Kearns-Sayre syndrome. Fascinatingly, the anaemia resolves and the level of the mtDNA deletion in blood falls (45,46). This explains why in terms of diagnosis, it is important to look in other tissues (muscle or uroepithelial cells) for pathogenic mtDNA variants. Previous studies have shown that the variant level in muscle tissue correlates well with the phenotype suggesting that the clinical features are, in part, driven by variant level (47).

#### Heteroplasmic mtDNA pathogenic variants with moderate selection in replicating tissues

Perhaps the best example is the common m.3243A>G mtDNA variant, which is associated with a very variable clinical phenotype ranging from severe MELAS to mild deafness or glucose intolerance (14,48–50). Patients show a definite decrease in m.3243A>G load in blood, with an initial steep fall over the first years of life and then a slower decline/stabilization (42,43). Therefore, variant levels in blood can be markedly different from urinary sediment cells or muscle (42,51). Large cohort studies of m.3243A>G cases revealed great phenotypic variation between individuals at the same variant level (42,52) and a recent study provided good evidence for the influence of nuclear genetic factors in clinical outcomes for m.3234A>G-related disease (50).

#### Heteroplasmic pathogenic variants with little or no selection in tissues

Good examples from this category are the m.8344A>G variant associated with myoclonic epilepsy and ragged-red fibres (MERRF), and the m.8993T>G/C causing Leigh syndrome or neurogenic weakness, ataxia and retinitis pigmentosa (53,54). These variants tend to have an even and stable level across all tissues (55,56), which in terms of diagnosis means they can be easily detected in blood. Also, the clinical phenotype for the m.8993T>G/C correlates much better with variant level than in the m.3243A>G variant (56).

### Treatment of mtDNA disease

Whilst there have been major advances in our understanding of mtDNA disease, there is much slower progress in terms of treatment. The care of patients has improved markedly over recent years. The development of large clinical cohorts in many countries has enabled the development of clinical guidelines. These include better surveillance regimes to detect clinical features early and select the best treatment options for acute presentations of mitochondrial disease. There are also many small molecule therapies being considered or in clinical trials (57), but there is currently no proven therapy for mtDNA disease.

**Figure 2:**
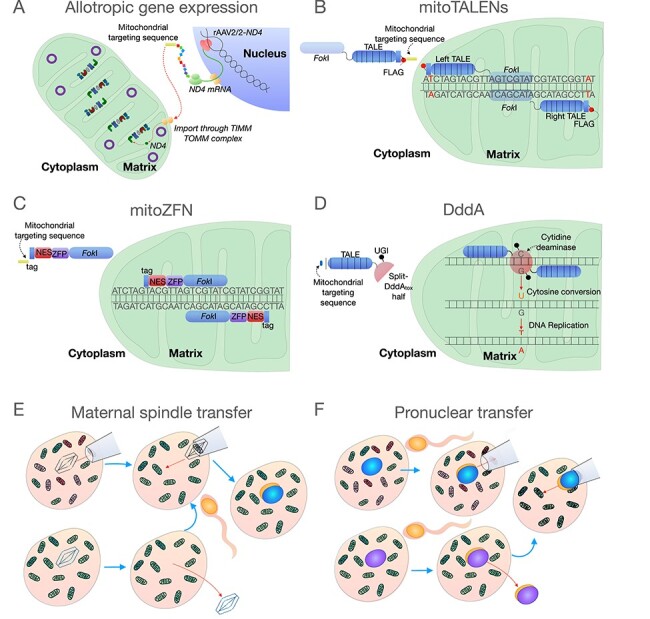
Strategies to rescue and prevent transmission of pathogenic mtDNA variants. (**A**) Allotropic gene expression has been used to rescue pathogenic variants of Leber’s Hereditary Optic Neuropathy using an adenoviral associated vector containing the MT-ND4 gene (rAAV2/2-ND4) with a mitochondrial targeting sequence. This allows the gene to be transcribed in the nucleus, translated in the cytosol, and later imported into the mitochondria to be incorporated into complex I. In theory, this could be applied to other mtDNA gene variants in the future. (**B**) MitoTALENs have been used to modulate the heteroplasmy of mtDNA single-nucleotide variants. These nucleases have a mitochondrial targeting sequence which allows them to be imported into the mitochondria. MitoTALENs work in pairs (left and right) which bind to mtDNA variant sequences, bringing the FokI nucleases into close proximity, which leads to cleavage of variant mtDNA molecules and subsequent degradation. (**C**) mitoZFNs, like mitoTALENS, have been used to modulate mtDNA variant heteroplasmy. The Zinc finger nuclease construct also has a mitochondrial targeting sequence for import into the mitochondria. Like with TALENS, the mitoZFNs bind to the mtDNA variant based on sequence-specificity, bringing the two FokI nucleases together and leading to cleavage of the mtDNA variant, which is degraded. (**D**) Ddda have been used to revert an mtDNA single-nucleotide variant to its wild-type state via cytosine conversion. A mitochondrial targeting sequence is used to import the construct into the mitochondria, where two TALE sequences locate the two halves of the DddA over the variant site. The Ddda enzyme converts cytosine to uracil. When the DNA is replicated this U-G pair is then converted to a T-A pair. (**E**, **F**) Mitochondrial donation for women with pathogenic mtDNA variants. Mitochondrial donation involves the transfer of the nuclear DNA from an zygote (E) or oocyte (F) from a woman with a pathogenic mtDNA variant into an enucleated, recipient donor zygote or oocyte with healthy mtDNA in order prevent the transmission of the pathogenetic mtDNA variant to the offspring. In pronuclear transfer (E) both donor and patient oocytes are fertilized and allowed to progress into the zygote, then the pronuclei of the donor zygote is removed and replaced by the pronuclei from the patient. In maternal spindle transfer (F), the metaphase II meiotic spindle of a donor oocyte is removed and replaced by the spindle from a mature oocyte of the patient followed by fertilization and development.

For many years, there has been an interest in correcting the genetic defect by gene therapy, which may seem easier to achieve than nuclear gene therapy, due to the misleading simplicity of the mitochondrial genome. However, the mitochondrial inner membrane, which is highly specialized for OXPHOS and impermeable to most molecules, including DNA, has been a major challenge. Nevertheless, several approaches have been considered and there has been substantial progress recently. Allotopic expression (Fig. 2A) involves delivering wild-type copies of the mutated mtDNA gene to the cytosol using viral vectors (58). This would be translated in the cytosol and the protein later imported by the mitochondria using a mitochondrial targeting sequence. Allotopic expression has been recently trialled in patients with the m.11778G>A variant in *MT-ND4*, which accounts for ~75% of LHON (59). In this trial, the viral vector was injected locally to deliver the *MT-ND4* to the affected retinal ganglion cells of one of the patients eyes. The authors reported sustained visual improvement in both eyes over a 96-week follow-up period suggesting that the viral vector DNA may have transferred between eyes.

Another approach is to manipulate variant level by targeting the pathogenic mtDNA variant. This was first attempted using peptide nucleic acid oligomers to block replication of mutated genomes (60,61). Initial reports using *in vitro* replication systems were encouraging, unfortunately uptake into mitochondria could not be confirmed and responses in cells were disappointing. Another approach is to use mitochondrially targeted endonucleases which will cross the mitochondrial membranes and specifically degrade pathogenic variants (62,63). However, known endonucleases could only target a limited number of pathogenic variants and could cross-talk with wild-type genomes, which led to the development of mitochondrially targeted programmable nucleases, namely the mitochondrial transcription activator-like effector nucleases (mitoTALENs) or mitochondrial zinc finger nucleases (mitoZFNs) (Fig. 2B and C). Using a mouse model of mtDNA disease (heteroplasmic m.5024C>T mt-tRNA^Ala^ mouse), both mitoTALENs and mitoZFNs were shown to significantly decreased the mutant load and improve the cells biochemical phenotype (62,63). There remain challenges in how this treatment could be translated into clinical practice for multisystemic diseases. Recently, the enzyme DddA has been reported to be capable of precise mtDNA editing (Fig. 2D), which represents a new strategy not yet fully explored (64).

### Understanding and preventing the transmission of pathogenic mtDNA variants

MtDNA disease is progressive in most patients and often leads to significant morbidity or early death. With no disease-modifying treatments available, a clear priority is trying to prevent the maternal transmission of pathogenic variants. This presents several challenges, and the development of new *in vitro* fertilization (IVF) techniques has improved the options available. When considering reproductive advice to women with pathogenic mtDNA variants there are several different factors to consider, and it is important that families are advised on their reproductive options by clinicians who are aware of the challenges and uncertainties. The three groups to consider are:

#### Homoplasmic pathogenic mtDNA variants

While these will be transmitted to all offspring, some of these variants show incomplete penetrance (e.g. the three common LHON variants), and transmission does not necessarily mean the offspring will be affected (65). It is likely that nuclear genetic factors will influence the expression of the disease (66), but these factors have not yet been identified, highlighting how challenging decisions can be for these families.

#### Single, large-scale mtDNA deletions and some other sporadic pathogenic mtDNA variants

Most cases of single, large-scale mtDNA deletion are sporadic and the risk of transmission to offspring is low. This suggests that deletions are selected against during the mitochondrial genetic bottleneck in oocyte development, which is supported by studies in mice showing that severe variants are not transmitted through the germline (23). Some sporadic, pathogenic, single-nucleotide variants at very low levels in blood may also not be readily transmitted through the bottleneck (16).

#### Most heteroplasmic mtDNA pathogenic variants

These variants are maternally transmitted through the germline bottleneck which results in variable variant levels in the offspring. However, the tightness of the bottleneck differs between variants (67–70). For example, the m.8993T>G/C has a tight bottleneck and segregates markedly, resulting in offspring with very high or very low variant levels. Others, such as m.8344A>G, show much less variation in oocytes and thus offspring. This information is extremely important in terms of genetic advice for families. What influences the bottleneck size and whether nuclear genetic factors impact this remains unknown (71).

#### Reproductive options for women with pathogenic mtDNA variants

There are many challenges when considering reproductive options for affected women and genetic counselling should be started early since fertility declines with age and options become more limited. Advice should be individualized for the patient’s circumstances and the different mtDNA variants. It is important to consider the mother’s health since there are increased risks during pregnancy for some women (72,73). Counselling should address all options including voluntary childlessness, adoption and oocyte donation. For women wishing to have biological children, reproductive options, depending on the variant, are prenatal testing, preimplantation genetic diagnosis (PGD) and mitochondrial donation (mitochondrial replacement therapy). However, the availability of these options is dependent on what is allowed/available in individual countries.

#### Prenatal diagnosis

Prenatal diagnosis involves testing the variant level in foetal tissue obtained by chorionic villus biopsy or amniocentesis (74,75). The variant level detected in this tissue reflects that seen in tissues of offspring and provides valuable information. However, if the level of variant is high, predicting severe disease, the only options are to go ahead with pregnancy, despite the risk, or to terminate the pregnancy. One of the major challenges is to decide the risk associated with a particular pathogenic variant at a specific level and this uncertainty can lead to an agonizing decision for parents. The risk of the procedure also needs to be addressed (around 1% loss of pregnancy).

#### Preimplantation genetic diagnosis

This involves testing embryos before implanting an embryo with low levels of variant (74,75) and is a good option for women affected by variants that widely segregate in oocytes (e.g. m.8993T>G/C), since some oocytes will inherit variant levels well below disease-threshold (70). For other heteroplasmic variants, the challenges of PGD are much greater. For example, for women with high levels of a variant with low germline segregation, there may be no embryos with variant levels below what is considered low risk of disease (71).

#### Mitochondrial donation

The rationale for developing these IVF techniques was to offer women, with homoplasmic variants or variants at high levels, the opportunity to have their own biological children with low levels of variant and therefore low risk of disease (76). The most experience with human embryos is with metaphase II spindle transfer (MST) and pronuclear transfer (PNT). MST involves the transfer of the metaphase II spindle from the oocyte with the pathogenic variant into a donor oocyte with normal mitochondria after removal of the donor’s spindle (Fig. 2E) (77). Following transfer, the oocyte is then fertilized. PNT is performed immediately after fertilization with the transfer of the pronuclei from the single cell zygote with the pathogenic mtDNA variant to the donor enucleated zygote with normal mitochondria (Fig. 2F) (78). Both techniques have been evaluated using human oocytes and shown that following transfer development to clinical grade embryos is feasible (76). As recently highlighted in the report from the USA Academies of Science and Medicine and the Royal Society (79), IVF regulation is very variable in different countries. Some countries have a highly regulated system, whereas in others there is little or no regulation. In the UK, IVF is regulated by the Human Fertilisation and Embryology Authority and, following the passing of the Mitochondrial Donation regulations through the UK Parliament, Mitochondrial Donation has been approved for clinical use under strict regulations (80).

## Conclusions

Since the finding of the first pathogenic variants over 30 years ago, there has been remarkable progress in our understanding of these diseases. Whilst basic studies have added hugely to our knowledge, the unique genetics of mitochondria has emphasized the important role of clinical genetic based research in improving the care of these patients. There remain many challenges ahead but the care pathways in place have significantly improved the lives of patients with mitochondrial disease.

Important studies in the future will inevitably focus of understanding disease mechanisms and using this knowledge to develop treatments. Unanswered questions include why mtDNA diseases get worse with time and whether this relates to the continued clonal expansion of variants? Why are there tissue specific differences in the phenotype of specific mtDNA variants? What is the role of nuclear factors in mtDNA disease? Can we cure mtDNA disease? Over the next decade, we are confident that significant progress will be made, and the care of patients will improve.

*Conflict of Interest statement*. D.M.T. reports personal fees from Khondrion, IMEL Biotherapeutics, Pretzel Therapeutics, Reneo Pharmaceuticals and Nanna Therapeutics, outside the submitted work. T.M.B.G, A.E.V., Y.S.N. and S.J.P. have no conflicts of interest to declare. All co-authors have seen and agree with the contents of the manuscript and there is no financial interest to report.
